# (2*S*,4*R*)-4-Ammonio-5-oxopyrrolidine-2-carboxylate

**DOI:** 10.1107/S1600536810004277

**Published:** 2010-03-13

**Authors:** Krzysztof Kaczmarek, Jakub Wojciechowski, Wojciech M. Wolf

**Affiliations:** a Institute of Organic Chemistry, Technical University of Łódź, ul. Żeromskiego 116, 90-924 Łódź, Poland; b Institute of General and Ecological Chemistry, Technical University of Łódź, ul. Żeromskiego 116, 90-924 Łódź, Poland

## Abstract

In the crystal structure of the title compound, C_5_H_8_N_2_O_3_, the mol­ecules exist in the zwitterionic form. The pyrrolidine ring adopts an envelope conformation with the unsubstituted endocyclic C atom situated at the flap. The other four endocyclic atoms are coplanar with the exocyclic carbonyl O atom, with an r.m.s. deviation from the mean plane of 0.06 Å. The carboxyl­ate substituent is located axially, while the ammonium group occupies an equatorial position. In the crystal structure, the mol­ecules are linked through N—H⋯O hydrogen bonds, forming a three-dimensional network.

## Related literature

For mol­ecular recognition in *N*-methyl amino acids and proline residues, see: Dugave & Demange (2003 ▶). For the construction of modified amino acids, see: Dumy *et al.* (1997 ▶); Keller *et al.* (1998 ▶); Mutter *et al.* (1999 ▶); Tuchscherer & Mutter (2001 ▶); Paul *et al.* (1992 ▶). For pyroglutamic acid derivatives, see: Zabrocki *et al.* (1988 ▶); Kaczmarek *et al.* (2005 ▶). For the preparation of the title compound, see: Kaczmarek *et al.* (2001 ▶); Kaczmarek (2009 ▶). For asymmetry parameters, see: Griffin *et al.* (1984 ▶).
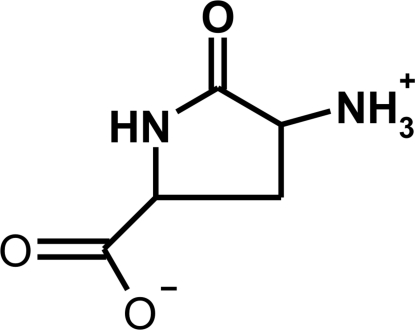


## Experimental

### 

#### Crystal data

C_5_H_8_N_2_O_3_
*M* *_r_* = 144.13
Orthorhombic, 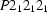

*a* = 5.9790 (3) Å
*b* = 9.3665 (4) Å
*c* = 11.3809 (5) Å
*V* = 637.36 (5) Å^3^
*Z* = 4
Cu *K*α radiation
μ = 1.08 mm^−1^
*T* = 293 K
0.40 × 0.40 × 0.10 mm

#### Data collection

Bruker SMART APEX diffractometer
Absorption correction: multi-scan (*SADABS*; Bruker, 2003 ▶) *T*_min_ = 0.707, *T*_max_ = 0.900
7227 measured reflections
1169 independent reflections
1168 reflections with *I* > 2σ(*I*)
*R*_int_ = 0.030

#### Refinement

*R*[*F* ^2^ > 2σ(*F* ^2^)] = 0.027
*wR*(*F* ^2^) = 0.069
*S* = 1.08
1169 reflections
125 parameters
H atoms treated by a mixture of independent and constrained refinement
Δρ_max_ = 0.13 e Å^−3^
Δρ_min_ = −0.17 e Å^−3^
Absolute structure: Flack (1983 ▶), 461 Friedel pairs
Flack parameter: 0.1 (2)

### 

Data collection: *SMART* (Bruker, 2003 ▶); cell refinement: *SAINT-Plus* (Bruker, 2003 ▶); data reduction: *SAINT-Plus*; program(s) used to solve structure: *SHELXTL* (Sheldrick, 2008 ▶); program(s) used to refine structure: *SHELXTL*; molecular graphics: *SHELXTL* and *Mercury* (Macrae *et al.*, 2008 ▶); software used to prepare material for publication: *SHELXTL* and *publCIF* (Westrip, 2010 ▶).

## Supplementary Material

Crystal structure: contains datablocks I, global. DOI: 10.1107/S1600536810004277/bt5187sup1.cif

Structure factors: contains datablocks I. DOI: 10.1107/S1600536810004277/bt5187Isup2.hkl

Additional supplementary materials:  crystallographic information; 3D view; checkCIF report

## Figures and Tables

**Table 1:** Hydrogen-bond geometry (Å, °)

*D*—H⋯*A*	*D*—H	H⋯*A*	*D*⋯*A*	*D*—H⋯*A*
N1—H1⋯O2^i^	0.82 (2)	2.07 (2)	2.8535 (15)	161.2 (18)
N2—H2⋯O1^ii^	0.88 (2)	1.87 (2)	2.7346 (16)	168.5 (17)
N2—H3⋯O1^iii^	0.897 (17)	1.886 (17)	2.7788 (14)	173.4 (17)
N2—H4⋯O2^iv^	0.868 (17)	1.935 (17)	2.7967 (15)	172.3 (17)

Symmetry codes: (i) 
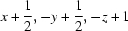
; (ii) 
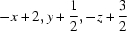
; (iii) 
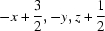
; (iv) 
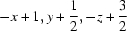
.

**Fig. 1.**
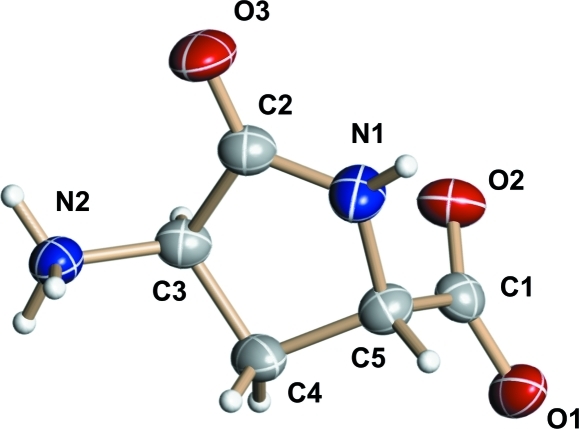
Molecule of the title compound. Displacement ellipsoids are drawn at the 50% probability level.

**Fig. 2.**
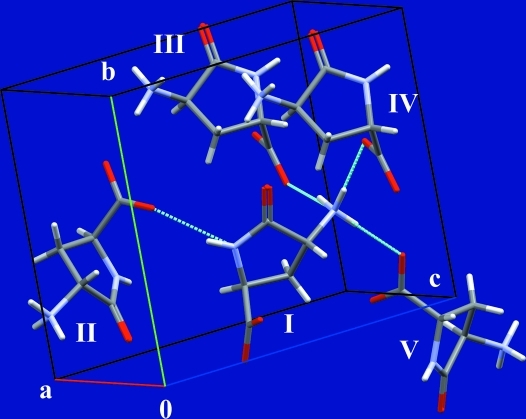
View of hydrogen bonding in the crystal of the title compound. Symmetry codes: I (x, y, z); II (x + 1/2,-y+1/2,-z+1); III (-x + 2, y + 1/2,-z+3/2); IV (-x + 1, y + 1/2,-z+3/2); V (-x + 3/2,-y, z + 1/2).

## supplementary crystallographic information

### Comment

*N*-methyl amino acids and proline residues in the peptide chain may cause the *cis-trans* isomerisation of the amide bond and lead to conformational changes, which influence the molecular recognition (Dugave & Demange, 2003). Importance of the *cis*-amide bonds for the peptide bioactivity led to the construction of modified amino acids, which could lock a peptide bond in the *cis*-geometry (Dumy *et al.*, 1997; Keller *et al.*, 1998; Mutter *et al.*, 1999; Tuchscherer & Mutter, 2001). In particular, Paul *et al.* (1992) designed mimetics of the cis-peptide bond based on the substitituted pyroglutamic acid residue. In contrast with a tetrazole replacement for the peptide bond, the pyroglutamic acid derivatives are more rigid (Zabrocki *et al.*, 1988). Their carboxylic group could be either donor or acceptor of hydrogen bond without invloving the polypeptide main chain amide moieties (Kaczmarek *et al.*, 2005).

The 4-aminopyroglutamic acid is a particularly useful residue for building the conformationally restricted peptide chains. Depending on the absolute configuration at both chiral centers it may be applied to construct the VIa or VIb β-turn mimetics.

The title compound may be obtained by two different methods elaborated by us, *i.e.* by electrophilic amination reaction of N-protected (S)-pyroglutamate ester, which gives separable 9:1 mixture of (2*S*,4R) and (2*S*,4S) diastereoisomers (Kaczmarek *et al.*, 2001) or through Michael addition of dehydroalanine derivatives to sodium salt of *N*-benzyloxycarbonylaminomalonate ester, which gives after hydrolysis and decarboxylation mixture of all four possible stereomers. The details of the last reaction and resolution of stereoisomers will be described elsewhere (Kaczmarek, 2009).

A view of the title compound is given in Fig. 1. The molecule has two chiral centres *viz*. C3 and C5. Their absolute configurations follow from the synthetic procedure and are *R* and *S*, respectively.

The pyrrolidine ring adopts an envelope conformation with N1, C2, C3 and C5 almost coplanar and the C4 situated at the flap.

Additionally, the former four endocyclic atoms are coplanar with the exocyclic carbonyl oxygen, the average r.m.s. deviation from the mean plane is 0.06 Å.

The three lowest ring asymmetry parameters (Griffin *et al.*, 1984) are: C_S_(C4) = 1.26 (14), C_2_(C2) = 11.92 (14), C_2_(N1) = 15.46 (14)°. The carboxylate substituent is located axially in conformation stabilized by the short N1···O2 contact [2.787 (2) Å], while the ammonium group occupies equatorial position.

In the crystal each molecule is linked through  N—H ···O hydrogen bonds with eight adjacent molecules, their deatils are shown in Table 2 and Fig. 2.

### Experimental

An optically pure (ee>99%) *N*'-benzyloxycarbonyl protected precursor of the title compound was hydrogenated in methanol solution over 10% palladium on charcoal, which resulted in precipitation of the final product. After filtration of solids final product was washed out of the catalyst with the aim of water. The (2*S*,4R)-4-aminopyroglutamic acid crystals were grown from this water solution by slow evaporation.

### Refinement

All H atoms were located in difference Fourier maps and refined freely.

### Crystal data

**Table:**

C_5_H_8_N_2_O_3_	*D*_x_ = 1.502 Mg m^−^^3^
*M**_r_* = 144.13	Melting point: 423(2) K
Orthorhombic, *P*2_1_2_1_2_1_	Cu *K*α radiation, λ = 1.54178 Å
Hall symbol: P 2ac 2ab	Cell parameters from 7056 reflections
*a* = 5.9790 (3) Å	θ = 6.1–70.8°
*b* = 9.3665 (4) Å	µ = 1.08 mm^−^^1^
*c* = 11.3809 (5) Å	*T* = 293 K
*V* = 637.36 (5) Å^3^	Prism, colourless
*Z* = 4	0.40 × 0.40 × 0.10 mm
*F*(000) = 304	

### Data collection

**Table:**

Bruker SMART APEX diffractometer	1169 independent reflections
Radiation source: fine-focus sealed tube	1168 reflections with *I* > 2σ(*I*)
graphite	*R*_int_ = 0.030
ω scans	θ_max_ = 70.8°, θ_min_ = 6.1°
Absorption correction: multi-scan (*SADABS*; Bruker, 2003)	*h* = −6→5
*T*_min_ = 0.707, *T*_max_ = 0.900	*k* = −11→11
7227 measured reflections	*l* = −13→13

### Refinement

**Table:**

Refinement on *F*^2^	Hydrogen site location: inferred from neighbouring sites
Least-squares matrix: full	H atoms treated by a mixture of independent and constrained refinement
*R*[*F*^2^ > 2σ(*F*^2^)] = 0.027	*w* = 1/[σ^2^(*F*_o_^2^) + (0.0468*P*)^2^ + 0.0681*P*] where *P* = (*F*_o_^2^ + 2*F*_c_^2^)/3
*wR*(*F*^2^) = 0.069	(Δ/σ)_max_ < 0.001
*S* = 1.08	Δρ_max_ = 0.13 e Å^−^^3^
1169 reflections	Δρ_min_ = −0.17 e Å^−^^3^
125 parameters	Extinction correction: *SHELXL97* (Sheldrick, 2008), Fc^*^=kFc[1+0.001xFc^2^λ^3^/sin(2θ)]^-1/4^
0 restraints	Extinction coefficient: 0.047 (3)
Primary atom site location: structure-invariant direct methods	Absolute structure: Flack (1983), 461 Friedel pairs
Secondary atom site location: difference Fourier map	Flack parameter: 0.1 (2)

### Special details

**Table:**

Geometry. All esds (except the esd in the dihedral angle between two l.s. planes) are estimated using the full covariance matrix. The cell esds are taken into account individually in the estimation of esds in distances, angles and torsion angles; correlations between esds in cell parameters are only used when they are defined by crystal symmetry. An approximate (isotropic) treatment of cell esds is used for estimating esds involving l.s. planes.
Refinement. Refinement of *F*^2^ against ALL reflections. The weighted *R*-factor wR and goodness of fit *S* are based on *F*^2^, conventional *R*-factors *R* are based on *F*, with *F* set to zero for negative *F*^2^. The threshold expression of *F*^2^ > σ(*F*^2^) is used only for calculating *R*-factors(gt) etc. and is not relevant to the choice of reflections for refinement. *R*-factors based on *F*^2^ are statistically about twice as large as those based on *F*, and *R*- factors based on ALL data will be even larger.

### Fractional atomic coordinates and isotropic or equivalent isotropic displacement parameters (Å^2^)

**Table:**

	*x*	*y*	*z*	*U*_iso_*/*U*_eq_	
O1	0.85679 (17)	−0.10762 (9)	0.57781 (8)	0.0373 (3)	
H2	0.838 (4)	0.3509 (18)	0.9130 (15)	0.042 (4)*	
O2	0.57914 (17)	0.04638 (10)	0.55325 (8)	0.0383 (3)	
O3	0.6519 (2)	0.46234 (9)	0.68254 (10)	0.0469 (3)	
N1	0.8566 (2)	0.27436 (11)	0.61237 (10)	0.0353 (3)	
H1	0.917 (4)	0.3094 (19)	0.5549 (17)	0.054 (5)*	
C1	0.7725 (2)	0.01571 (13)	0.58351 (10)	0.0283 (3)	
C5	0.9163 (2)	0.12935 (13)	0.64357 (11)	0.0304 (3)	
H51	1.078 (3)	0.1140 (16)	0.6258 (13)	0.032 (4)*	
C4	0.8646 (3)	0.12504 (13)	0.77672 (11)	0.0340 (3)	
H41	0.807 (3)	0.0354 (17)	0.8009 (15)	0.045 (5)*	
H42	0.995 (4)	0.156 (2)	0.8147 (18)	0.056 (5)*	
C3	0.6848 (2)	0.23810 (12)	0.79144 (11)	0.0297 (3)	
H31	0.541 (3)	0.1976 (15)	0.7877 (14)	0.030 (4)*	
N2	0.7038 (2)	0.31400 (11)	0.90532 (10)	0.0310 (3)	
H4	0.607 (3)	0.3822 (18)	0.9140 (14)	0.036 (4)*	
H3	0.695 (3)	0.249 (2)	0.9630 (14)	0.042 (4)*	
C2	0.7255 (2)	0.34135 (13)	0.68928 (11)	0.0319 (3)	

### Atomic displacement parameters (Å^2^)

**Table:**

	*U*^11^	*U*^22^	*U*^33^	*U*^12^	*U*^13^	*U*^23^
O1	0.0425 (6)	0.0262 (4)	0.0432 (5)	0.0027 (4)	−0.0079 (4)	−0.0062 (4)
O2	0.0334 (6)	0.0347 (5)	0.0466 (5)	−0.0016 (4)	−0.0093 (4)	0.0089 (4)
O3	0.0561 (7)	0.0309 (5)	0.0536 (6)	0.0133 (5)	0.0046 (5)	0.0104 (4)
N1	0.0451 (7)	0.0234 (5)	0.0374 (6)	−0.0044 (5)	0.0072 (5)	0.0050 (4)
C1	0.0331 (7)	0.0267 (6)	0.0251 (5)	−0.0021 (4)	0.0001 (5)	0.0034 (4)
C5	0.0311 (7)	0.0241 (6)	0.0361 (6)	−0.0008 (5)	0.0004 (5)	0.0004 (5)
C4	0.0435 (8)	0.0250 (6)	0.0335 (6)	0.0040 (5)	−0.0074 (6)	0.0002 (5)
C3	0.0294 (7)	0.0257 (5)	0.0340 (6)	−0.0027 (5)	−0.0020 (4)	0.0037 (5)
N2	0.0327 (7)	0.0257 (5)	0.0345 (5)	0.0022 (5)	0.0027 (4)	0.0029 (4)
C2	0.0321 (7)	0.0270 (6)	0.0367 (6)	−0.0015 (5)	−0.0022 (5)	0.0044 (5)

### Geometric parameters (Å, °)

**Table:**

O1—C1	1.2621 (16)	C4—C3	1.5187 (19)
O2—C1	1.2399 (17)	C4—H41	0.949 (17)
O3—C2	1.2179 (16)	C4—H42	0.94 (2)
N1—C2	1.3321 (17)	C3—N2	1.4826 (16)
N1—C5	1.4485 (15)	C3—C2	1.5318 (16)
N1—H1	0.82 (2)	C3—H31	0.939 (17)
C1—C5	1.5295 (17)	N2—H2	0.88 (2)
C5—C4	1.5470 (17)	N2—H4	0.867 (18)
C5—H51	0.997 (17)	N2—H3	0.898 (18)
			
C2—N1—C5	115.19 (10)	C3—C4—H41	109.0 (11)
O3—C2—N1	127.55 (12)	C5—C4—H41	112.3 (10)
O3—C2—C3	125.30 (12)	C3—C4—H42	108.7 (13)
N1—C2—C3	107.15 (11)	C5—C4—H42	106.1 (13)
N1—C5—C1	113.86 (10)	H41—C4—H42	116.4 (17)
N1—C5—C4	102.44 (10)	N2—C3—C4	112.11 (10)
C1—C5—C4	107.90 (10)	N2—C3—C2	110.40 (10)
C3—C4—C5	103.37 (10)	N2—C3—H31	107.7 (9)
C4—C3—C2	104.12 (10)	C4—C3—H31	111.1 (9)
C2—N1—H1	126.6 (13)	C2—C3—H31	111.5 (9)
C5—N1—H1	117.8 (13)	C3—N2—H2	110.3 (11)
O2—C1—O1	124.76 (12)	C3—N2—H4	113.6 (11)
O2—C1—C5	119.14 (11)	H2—N2—H4	107.9 (16)
O1—C1—C5	115.82 (11)	C3—N2—H3	108.0 (11)
N1—C5—H51	108.9 (9)	H2—N2—H3	104.4 (16)
C1—C5—H51	110.7 (9)	H4—N2—H3	112.3 (15)
C4—C5—H51	112.9 (8)		
			
N1—C5—C4—C3	26.38 (13)	O2—C1—C5—N1	−26.96 (16)
C5—C4—C3—C2	−25.98 (13)	O1—C1—C5—N1	158.87 (11)
C5—N1—C2—O3	−179.48 (14)	O2—C1—C5—C4	86.02 (13)
C5—N1—C2—C3	1.41 (16)	O1—C1—C5—C4	−88.15 (13)
C4—C3—C2—N1	16.23 (14)	C1—C5—C4—C3	−94.06 (11)
C4—C3—C2—O3	−162.91 (14)	C5—C4—C3—N2	−145.33 (10)
C2—N1—C5—C4	−17.99 (15)	N2—C3—C2—O3	−42.41 (18)
C2—N1—C5—C1	98.23 (14)	N2—C3—C2—N1	136.73 (12)

### Hydrogen-bond geometry (Å, °)

**Table:**

*D*—H···*A*	*D*—H	H···*A*	*D*···*A*	*D*—H···*A*
N1—H1···O2^i^	0.82 (2)	2.07 (2)	2.8535 (15)	161.2 (18)
N2—H2···O1^ii^	0.88 (2)	1.87 (2)	2.7346 (16)	168.5 (17)
N2—H3···O1^iii^	0.897 (17)	1.886 (17)	2.7788 (14)	173.4 (17)
N2—H4···O2^iv^	0.868 (17)	1.935 (17)	2.7967 (15)	172.3 (17)

Symmetry codes: (i) *x*+1/2, −*y*+1/2, −*z*+1; (ii) −*x*+2, *y*+1/2, −*z*+3/2; (iii) −*x*+3/2, −*y*, *z*+1/2; (iv) −*x*+1, *y*+1/2, −*z*+3/2.
